# A hill tribe community advisory board in Northern Thailand: lessons learned one year on

**DOI:** 10.1186/s12939-024-02323-z

**Published:** 2024-11-18

**Authors:** Carlo Perrone, Nipaphan Kanthawang, Phaik Yeong Cheah

**Affiliations:** 1 Mahidol-Oxford Tropical Medicine Research Unit, Faculty of Tropical Medicine, Mahidol University, Bangkok, Thailand; 2 Centre for Tropical Medicine and Global Health, Nuffield Department of Medicine, University of Oxford, Oxford, UK

**Keywords:** Community-based research, Community advisory board, Community engagement, Health disparate minority and vulnerable populations

## Abstract

Northern Thailand and its neighbouring regions are home to several minority ethnic groups known as hill tribes, each with their own language and customs. Hill tribe communities live mostly in remote agricultural communities, face barriers in accessing health, and have a lower socio-economic status compared to the main Thai ethnic group. Due to their increased risk of infectious diseases, they are often participants in our research projects.

To make sure our work is in line with the interests of hill tribe communities and respects their beliefs and customs, we set up a hill tribe community advisory board. We consult the members before, during, and after our projects are carried out. This manuscript recounts how we set up the community advisory board and our reflections following one year of activities. Our experience strongly supports engaging with community advisory boards when working with minority ethnic groups in lower and middle-income settings. In particular, we found that over time, as researchers and members familiarise with one another and their respective environments, exchanges gain meaning and benefits increase, stressing the advantages of long-term collaborations over short or project-based ones.

## Background

The Chiang Rai Clinical Research Unit (CCRU), part of the MORU Tropical Health Network, was set up in 2013 in the provincial capital of Chiang Rai, Northern Thailand. CCRU conducts research and community projects on infectious diseases prevalent in this province such as scrub typhus [1]. The Chiang Rai province in Thailand is nestled between Myanmar and Laos, with striking lush mountains running north-to-west and fertile fields stretching out east to the Mekong river. For decades, the province has been the cross-roads of peoples and cultures, and is home to several ethnic minority groups. Approximately 20% of the 1.3 million people living in Chiang Rai province are from hill tribe ethnic groups. The main hill tribe ethnic groups are Hmong, Akha, Lahu, Lisu, Karen, and Yao. Hill tribe communities usually live in rural and remote parts of the province. Because of this and due to their relatively low socio-economic status, they are at high risk of infectious diseases and are therefore common participants to our clinical research projects.

Researchers, healthcare workers, ethics committees and others who are not from these hill tribe communities are unable to fully grasp the cultural, moral, and ethical frameworks of hill tribe communities, making it difficult to judge whether our activities are in line with them. In addition, from our own qualitative work, we found that hill tribe members often faced challenges accessing healthcare and understanding research information due to language barriers and uncertain legal status [2, 3]. The hill tribe languages do not have written forms. These barriers lead to inequities in healthcare access and research participation.

To try and overcome these challenges, we established a hill tribe community advisory board (CAB), modelled after the long-standing Tak Province Community Ethics Advisory Board (T-CAB) facilitated by the MORU Tropical Health Network [4, 5].

## Main text

CABs comprising of community members, or variants, are the most widely documented structures for supporting community engagement [5–7]. The T-CAB, which was established in January 2009, comprises Burmese and Karen migrants living along the Thai-Myanmar border. They advise researchers on health research and health programmes on the Thai-Myanmar border.

Prior to this the establishment of the hill tribe Chiang Rai CAB (CR-CAB), the following activities were undertaken. Firstly, we observed a T-CAB meeting to understand the running of the meeting. Secondly, we conducted in-depth interviews with the T-CAB members and facilitators to understand the main roles of the T-CABs and the challenges encountered. Thirdly, we interviewed hill tribe leaders, healthcare workers, and community representatives in the main district (Mueang in Thai) of Chiang Rai province. Interviewees were selected from villages and primary care units we had collaborated with and where a high proportion of hill tribe communities resided. We started the interviews by explaining the potential scope of a CAB and asked interviewees if they thought it could be useful to communities and researchers locally. We also had a series of internal team meetings to discuss how the CAB would complement our existing community engagement initiatives.

Responses from the interviews were positive. The interviewees helped us identify potential CAB members, providing us contacts of people who had a hill tribe background or worked closely with hill tribe communities and they thought would be a good fit. In line with their recommendations, we invited those who could communicate in one or more hill tribe languages as well as Thai, were able to read, write, and communicate in Thai, and were respected figures in their communities. From the list of potential members, we made a shortlist trying to include the widest-range of roles and ethnicities while maintaining gender balance. We now have a total of fifteen members (a number chosen based on our experience with the T-CAB and other CABs we facilitate, to guarantee sufficient attendees in case of absences yet not enough to hinder productive discussions), from seven ethnic groups (Akha, Lahu, Hmong, Karen, Lisu, Mien, and Thai). Seven are female and eight male, with ages ranging between 42 and 68 years. The individuals originate from several districts within the Chiang Rai province. The distance between their locations and the usual meeting venue ranges from 12 to 83 km. We initially wished to recruit mostly farmers or day labourers from communities but were advised not to, as communication would have been too challenging, i.e. requiring translations to and from each hill tribe language, and throughout the year they would have been too busy with seasonal agricultural work to attend meetings.

Our first meeting was held in September 2023, and we have been meeting monthly since then. Meetings typically last 2.5 to 3 h and are held on Friday mornings, following the advice of our members. They take place in the city of Chiang Rai at the Hill Tribe Museum and Education Center [8]. Meetings start with a summary of the meeting from the previous month, followed by one or more presentations on past, current, or planned studies and related questions to and from CAB members, in this stage CAB members also raise their advice or opinion on presented studies, followed by a coffee break and then a round table where members discuss and share experiences and advice on the various issues (health-related or not) affecting their communities. Questions to the CAB may be general (e.g. “do you understand this project?” “what is not clear?”), or detail-oriented (e.g. if one particular sentence or figure is clear or not, who could be contacted to record materials in Lahu, where can information on a specific disease be found, which medium would be the best to provide health informatiton). Similarly, questions from the CAB related to presented projects projects may be general: (e.g. “after conducting research, where will the results be sent?”; or specifically related to presented projects “can we have access to mental health information or psychological counseling techniques?”; “does Covid-19 vaccine lead to blood clots?”). Meetings are carried out in Thai or Northern Thai dialect with translations to and from English, if presenters do not understand Thai. Because our team speaks Thai, there are rarely communication or language difficulties, but when they arise they are rapidly solved with the help of CAB or CCRU members. At the end of the meetings, details of the next meeting are discussed, followed by a communal lunch. Members are compensated 500 THB (about 14 USD) plus transportation (about 8–14 USD, depending on distance to the meeting venue). This corresponds to the daily wage of a semi-skilled labourer plus the average local cost of fuel to and from the venue.

With each meeting, CAB members and our team got increasingly familiar with each other and more comfortable asking each other questions. Researchers approach the CAB members for advice on aspects of their work; from study design to strategies for result dissemination and, particularly, recruitment and the informed consent process. The critical feedback provided has helped avoid several pitfalls. CAB members ask the opinion of the CCRU team on health-related news, they relay questions their communities have, and ask for advice on how to answer them.

We set up a mobile-phone chat initially intended to communicate about meeting logistics, upload meeting minutes, and CAB-related communications. Over time it has grown into a platform for sharing news, details of events of interest, and health-related messages among CAB members as well as a tool for rapid CAB consultation and asking for advice or support.

Since its launch, the CR-CAB has reviewed and suggested changes to the participant information materials for all research conducted by CCRU, including some studies from other parts of the MORU network. For example, they noted that using teaspoons to indicate the amount of blood drawn in studies, as recommended by Western and Thai ethics committees, is not appropriate because hill tribe communities do not normally use teaspoons. The members also aided in developing a communication strategy to convey sensitive hepatitis B and C laboratory results to 1,405 participants of a household survey. Additionally, the members helped identify 15 individuals who speak five non-Thai languages to be contacted as translators or interpreters for future health research. They also assisted in coordinating the translation of a video that will be used to explain a clinical trial to potential participants. With the help of the CR-CAB, culturally inappropriate or insensitive language and concepts, as well as examples communities could not understand or relate to, were avoided. The CAB reviewed the questionnaire for a mental health project to be carried out in 48 schools near the Thai-Myanmar and Thai-Laos borders. The members suggested alternatives for terms their communities would struggle with, recommended incorporating instructions before certain sections or questions, proposed formatting and question-order edits, and highlighted that in hill tribe communities, the family head may fill out forms for their family members. They also stressed the importance of feeding the survey results back to the communities, as this is usually not done. A tabular summary of the meeting topics, CAB feedback, and news from communities are given in Table 1.

**Table 1:** Summary of presentations, discussions, feedback provided, and community concerns reported by CAB members at each of the CAB meetings

Month and year; Main topics	Type of project	Feedback given and main questions asked about project	General community concerns and current health issues
September and October 2023 - Introduction to clinical research - Discussion on meeting time and location - Discussions about CR-CAB charter (CR-CAB aims, roles, membership, meeting format, communication, and documentation), expectations and suggestions	N/A	Meeting time and location agreed (mornings, 3rd Friday of each month, at the hill tribe museum, Chiang Rai). Chairperson and secretary elected. Expectations of researchers by CAB members: - Returning research results to communities and ensuring long-term commitment from CCRU to the CR-CAB - Researchers to respect local wisdom, practices, and culture (e.g. not plan activities on Christian holidays, as many hill tribe members are Christians and not Buddhists) - Respectful designations for hill tribes (e.g. Lisu or Akha) to be preferred to ones considered denigratory (e.g. “Muser” or “Ee-gaw” )	- Language barriers among hill tribe communities - Lack of familiarity with research among these communities
November 2023 “Causes of acute undifferentiated fever and the utility of biomarkers in Chiangrai”	Presentation of research findings	- Suggestion: Hill tribe interpreters should be trained and used for recruiting participants, focus should be on research and medical terminologies. Information materials in hill tribe languages should be provided, - Explanation asked on disease manifestations (e.g. how to differentiate leptospirosis and dengue fever)	- Lack of translation/knowledge on research in ethnic language. - Challenges in the informed consent - Fever is a common concern, there are traditional practices for treating fever among hill tribe communities, they are often used by community members - Conjunctivitis outbreak - Dengue and mosquito control awareness - Mental health and drug addiction
December 2023 “Prevalence of and factors associated with scrub typhus exposure among the hill tribe population living in high incidence areas in Thailand: a cross-sectional study”	Presentation of research findings	- Explanation asked on scrub typhus and whether researchers have provided information to communities at risk	- Influenza outbreak in the community - Villagers believe Covid-19 vaccine causes illness - Non-communicable diseases, are common - Some community members use traditional herbal remedies for NCD rather than pharmacological ones, which are recommended by the government - Communities worried about the health consequences of limestone mining in the area
January 2024 “School and community-based intervention for early detection of post-COVID mental health and long-term effects in Thai border schools: combined geospatial approach for network of support and policy Implications”	Planned research study, review of study documentation	- Members interested in attending mental health workshop - Members advised on the questionnaire, participant information sheet, informed consent form format, language and process	- Air pollution from bush fire - Infectious diseases (Influenza, Dengue) - Covid-19 vaccine misconceptions and concerns
February 2024 Overview of disease burden in rural South and Southeast Asia : A cross-sectional household health survey with questionnaire interviews and selected laboratory tests	Presentation of research findings, advice sought on how to feed individual viral hepatitis results back to participants	-Advised on how to approach illiterate individuals - prepare standard question to state who they would like to read study results to them - Confidentiality issues (primary care workers are accepted figures to provide results) - Advised on interview techniques - Advised how to share blood results to individuals (i.e. avoid “test negative” in favour of “you do not have the disease”)	- Concerns about Covid-19 vaccine side effects - Narcotics abuse - Air pollution - Health related to infectious disease, NCDs - Perceptions of receiving medicine from private and government hospitals
March 2024 Conversation preparedness for future pandemics with CR-CAB	Ongoing research/ engagement project	In case of a future pandemic: - Better resource management - Clear and consistent information from easily identifiable sources - There should be two-way communication - There should be strategies to reach ethnic minority groups, especially elderly and those in remote areas, those with language barriers, have electricity and communications access Experience during the COVID-19 pandemic: - Poor communication caused isolation, ostracism, unjustified blame; strict measures caused shortages including food and medicine, which lead to use of unorthodox treatments including Opium. - Guidelines on funerals or other religious practices were vague.	- Covid-19 vaccine side effects
April 2024 The challenges and potential solutions of achieving meaningful consent amongst research participants in northern Thailand: a qualitative study.	Presentation of research findings, suggestions on how to improve comprehension of participant information	*Mostly from first-hand experience of CAB members who acted as interpreters (formally and informally)* -Suggested sources of trained hill tribe interpreters available in Chiang Rai province - Training hill tribe interpreters rather than having relatives of participants as informal interpreters - Informal interpreters who speak Thai but cannot read should be avoided as translations will be inaccurate - Advised explanation of research project technique to target populations - If a reliable translator is not available in person, translations by telephone should be favoured over relatives - Probe comprehension rather than asking if participants understood the information presented or not	- Language barriers of hill tribe communities, education affects the quality of translation - Narcotic drug use, commerce, and related issues, violence - Natural disasters such as drought and wildfires
May 2024 Quick and easy scrub typhus diagnostic tools	Planned research study, advice sought on how to perform informed consent	- Advised about how to obtain informed consent and review participant information sheet and informed consent form - Advised to translate disease name that does not exist in hill tribe language into Thai or use images	- Road traffic accidents - Narcotic drug use and trafficking - Challenges of non-Thai citizen accessing to healthcare facilities - Concern about Covid-19 vaccine side effects - Natural disasters (lightning, drought) - Infectious diseases (Dengue fever) - Mental health issues e.g. depression, suicide
June 2024 Effects of a smartphone application for children and Family self-management on eating and physical activity behaviors among overweight late school-aged children.	Past research study	- Positive feedback and interest on the potential use of the app	- Dengue and mosquito prevention - Hypercholesterolaemia and non-communicable diseases - In one community, community and religious support helped individuals with drug addiction - Air quality improved but upper airway infections persist - Scamming (fraudulent calls to obtain bank account details) - Heathy diet promotion activities started by PCUs to battle increase in fast- and processed food consumption
July 2024 1. Guidelines for a poster on clinical research: obtaining consent and assent in children 2. Vulnerability and agency in research participants’ daily lives and the research Encounter: A qualitative case study of participants taking part in scrub typhus research in northern Thailand. 3. Covid vaccine safety	1. Ongoing project to improve the ethics of enrolling children in research 2. Presentation research findings 3. Presentation of relevant recent literature	Advised/revised poster translation, - e.g. “what will happen if we refuse to participate?” was found to sound threatening in Thai The presentation was appreciated, members wished to relay the information to their communities	- Outbreaks of dengue fever, influenza, hand-foot-mouth disease - Vulnerable groups have faced language barriers which cause delay accessing to healthcare - Concerns about Covid-19 vaccine side effect - Narcotic drug use and trafficking - Increased e-cigarette use among youths
August 2024 Co-creating information materials with communities to improve the Informed Consent process. (special meeting)	Planned research study, CAB asked to review study documentation	Revised wording of Participant Information Sheet and Informed Consent Form	- Outbreaks of dengue fever, influenza, hand-foot-mouth disease - Natural disaster e.g. flood, landslides, could cause mental health problems - Concern about Covid-19 vaccine side effects - Narcotic drug use and trafficking Community want to know more about HPV vaccine and monkeypox disease
October 2024 A community health podcast on understanding, preventing and managing scrub typhus	Planned research study, CAB asked to review study documentation	- Revised word used in Participant Information Sheet, Informed Consent Form and questionnaire	- Natural disasters (e.g. flood, landslide), could cause mental health disturbances - Dengue fever and common cold cases still occurring - Road traffic accidents

The CR-CAB activities are being evaluated through self-administered questionnaires completed by members after each meeting, as well as through informal discussions and team debriefs. In the questionnaires, members rated the topics presented as very important (highest level of a three point scale) for their community 79% of the time and quite important (middle level of a three point scale) 21% of the time. When asked to estimate what they had learned, 89% of respondents selected “a lot” (corresponding to the highest level of a three-point scale) and 96% stated that they planned to share their new knowledge with others. Aside from formal evaluation, the topics that elicited most interest were related to COVID-19, childhood obesity, and mental health among school children.

Attendance at the time of writing averages 78%, despite some members having to drive almost an hour each way to attend meetings. To date, none of the initial members have left the CAB. All those who attended the meetings as presenters and observers said the experience would benefit their future work and they expressed interest in attending other CAB meetings in the future. Of those presenting studies yet to be implemented or specific questions relating to ongoing studies or other procedures, all adapted their strategies or documents as advised by the CAB. When asked what they appreciated the most, learning about the culture and practices of the ethnic groups and the challenges they face was listed by 80% of presenters.

CAB members appreciated learning and sharing about health issues in other parts of the district, gaining more knowledge about infectious diseases and their prevention, research projects and their benefits, and relaying the information to their community; they also suggested directing future efforts to mental health, climate-related diseases, and non-communicable diseases.

We encountered some difficulties in establishing and implementing the CAB. Explaining the potential roles and functions of a CAB to prospective members was challenging, as the concept was unfamiliar to them. During the first few meetings, when members were not yet well-acquainted, facilitators had to make considerable efforts to encourage participants before obtaining feedback. Members also needed time to familiarize themselves with research concepts. However, these obstacles gradually disappeared over time. Also, the members of this CAB cover roles of relevance in their communities, and are not farmers or labourers, who represent the majority of the population. This choice was made to ease communication at the meetings, as minority ethnic groups with less educated backgrounds are unlikely to speak Thai. However, we need to recognise the risk of elitism which may be exacerbated by belonging to the CAB, which could lead to members eventually not being able to adequately represent their communities and compromise the CAB’s function.

Another challenge we are mindful of, is the risk of members developing views close to those of the researchers or feeling pressure to agree with researchers, leading to a lack of independence [9]. To minimise this risk, we are keeping the CAB as a long-term core activity rather than one funded from any specific project and are trying to keep team members responsible for CAB activities independent from those leading and carrying out clinical research, who usually attend as presenters or guests and do not hold any official positions.

As we aim to run community-led projects, we will also need to find the right balance between providing sufficient training to ensure ethical standards are followed, maintain the independence of the communities, and make sure CAB members are not instrumentalised to mirror the interests of researchers.

Despite these challenges, we have found that the CAB has proven to be an invaluable tool, not only for improving communication with participants but also for better understanding the health concerns of communities and the challenges they face in everyday life. It helps us put our work into perspective and regularly reminds us of our duties as researchers to the communities we work with, ensuring that we align with their needs and share our findings appropriately. In contexts where language, geography, or logistics create distance between researchers and communities, CABs serve as a means to meet and socialize in an informal setting where power dynamics are more balanced. After the initial work required to establish the CAB, the resources needed are minimal compared to the benefits.

## Conclusions

Our experience shows that time and sustained interactions are key because community members cannot be expected to advise ‘ad hoc’ on topics they know little of. Researchers have found that these ‘working relationships’ are crucial in engagement with community members [10, 11]. Over time, CR-CAB members become familiar with research, and researchers become familiar with the communities. Mutual respect and connections are built, and accountability, trust, and empowerment grow almost spontaneously. Consequently, the returns on investments in CABs will be exponentially greater the longer the CAB is in operation. We believe that governmental, non-governmental, and funding organizations should prioritize long-term commitments over one-off or project-based community consultations. This is especially important where research literacy is low, as is often the case in low- and lower-middle-income settings.

In the future, we will have to safeguard the independence of the CAB from any research project, including those led by members of our unit, and make sure increasing exposure to research as CAB members will not erode any ties to the communities. We also aim to encourage researchers from other organizations working in the province to consult with the CR-CAB. As a first step, we have informed them that the CAB meets monthly and have invited them to attend our meetings as observers. Additionally, we plan to consult CR-CAB members on health research priority setting, both to inform our work and to benefit other researchers in the region. Including the voices of under-reached communities in this process is rare but increasingly encouraged to address health inequities [12]. Much research has examined the role of CABs in high-income countries but less is known about CABs in low- and middle-income countries [7]. We will share our experiences from engaging with the CR-CAB, particularly with other CAB facilitators in low- and middle-income settings who may encounter similar challenges.

## Data Availability

The transcripts of the interivews, meeting minutes, and findings of the evaluation will be made available in anonymized forms upon reasonable request to the MORU Data Access Committee.

## Abbreviations

CAB: Community advisory board
CCRU: Chiang Rai Clinical Research Unit
CR-CAB: Chiang Rai Community Advisory Board
T-CAB: Tak Province Community Ethics Advisory Board
